# Influence of Silica Modulus on the Activation of Amorphous Wollastonitic Hydraulic Binders with Different Alumina Content: Study of Hydration Reaction and Paste Performance

**DOI:** 10.3390/ma17133200

**Published:** 2024-06-30

**Authors:** Mónica Antunes, Rodrigo Lino Santos, Ricardo Bayão Horta, Rogério Colaço

**Affiliations:** 1 Instituto Superior Técnico, University of Lisbon, Av. Rovisco Pais, 1049-001 Lisboa, Portugal; monica.h.antunes@tecnico.ulisboa.pt; 2 IDMEC—Instituto de Engenharia Mecânica, University of Lisbon, 1049-001 Lisboa, Portugal; 3 CIMPOR—Serviços, S.A., Avenida José Malhoa, nº 22—Floors 6 to 11, 1099-020 Lisboa, Portugal

**Keywords:** silica modulus, sodium silicate activation, amorphous wollastonitic binders, hydration reaction, paste properties

## Abstract

This study investigates how different sodium silicate SiO_2_/Na_2_O MS ratios (0.75, 0.9, and 1.2) affect the hydration behavior of amorphous wollastonitic hydraulic (AWH) binders containing various amounts of Al_2_O_3_ content (4, 7, 10, and 12%wt). The effects of and interaction between the MS ratio of the activator and the Al_2_O_3_ content of the sample on the hydration reaction and paste performance were investigated. The reaction was followed by calorimetry, and the pastes’ compressive strength performances were tested at different curing times (2, 7, and 28 days). The hydrated pastes were characterized by FTIR, thermogravimetry analysis, and X-ray diffraction. The calorimetric results show that a higher Al_2_O_3_ cContent and a higher MS ratio result in a longer induction period. In terms of paste performance, an increase of the Al_2_O_3_ coupled with an activation with a 1.2 MS ratio results in a lower compressive strength after 28 days of hydration; the results range from 76 to 52 MPa. A decrease of the MS ratio to 0.9 allowed the obtention of a narrower range of results, from 76 to 69 MPa. Even though a decrease of the MS ratio to 0.75 led to higher hydration kinetics and high compressive strength results at early ages, at 28 days of curing, a decrease in compressive strength was observed. This may be a consequence of the fast kinetic of the mixture, since the rapid growth of hydration products may inhibit the dissolution at later ages and increase the porosity of the paste. Moreover, the high Al intake in the hydration product, facilitated by the high sodium content of the activator, promotes the formation of a higher number of calcium aluminate silicate hydrate structures (C-A-S-H) to the detriment of calcium silicate hydrate structures (C-S-H), decreasing the compressive strength of the samples. The TGA results indicate that the samples hydrated with the MS075 solution resulted in a higher number of hydrated products at early ages, while the samples hydrated with the MS09 and MS1.2 solutions exhibit a steady increase with curing time. Hence, an equilibrium in the hydration kinetic promoted by Si saturation–undersaturation appears to be fundamental in this system, which is influenced by both the MS ratio and the Al(OH)^4−^ content in solution. The results of this study suggest that for this type of binder, optimal performance can be achieved by decreasing the MS ratio to 0.9. This composition allows for a controlled kinetic and overall higher compressive strength results in pastes produced with this AWH precursor.

## 1. Introduction

Since the raw materials used in ordinary Portland cement (OPC) production are economically accessible [1], cement plants are typically located near limestone quarries to avoid the need for the transportation of raw materials across larger distances [1,2]. However, variations in the Earth’s crust can lead to changes in the raw materials’ chemical composition, namely their aluminum, iron, and magnesium oxide content [3]. These changes in the composition of the raw materials affect the composition of the produced clinker and may result in changes in its performance [4,5,6,7]. Hence, it is important to study the influence of these variations when considering the large-scale production of a binder [3].

In this work, the studied binder is an amorphous wollastonitic hydraulic (AWH) binder, a slag-like binder that has been investigated as an OPC alternative due to its lower CO_2_ production emissions [8]. Previous works reported a competitive compressive strength in pastes when hydrated with a sodium silicate solution [9,10]. Until now, the binder production maintained a specific chemical composition, with a low content of Al_2_O_3_, MgO, and Fe_2_O_3_ elements (<2%wt). However, due to the known variability of natural raw materials, this study focused on the variation of Al_2_O_3_ content that can be incorporated into the clinker without compromising its performance, within the range of 4–12%wt.

The Al_2_O_3_ content also influences the hydration reaction of the binder by acting as a network former and a network modifier [11,12,13]. Typically, Al replaces Si in bridging position [14] and leads to an increase in the mean chain length in the hydrated product [14,15]. This chain length can increase from 3–5 tetrahedra, calcium silicate hydrate (C-S-H) of OPC, to 11 tetrahedra, as observed in the calcium aluminate silicate hydrate structures (C-A-S-H) of alkali-activated materials [14]. The charge imbalance caused by the Si^4+^ ⇔ Al^3+^ replacement is compensated by Na^+^ incorporation [14]. Nevertheless, in systems with a Ca/(Si + Al) ratio above 0.9 and under suitable activation conditions [14,15], replacement in tetrahedral positions is also possible [14,15]. The final hydrated material consists of a mixture of Si-O-Si and Si-O-Al bonding with a structure that ranges from semi-crystalline to amorphous [7].

Due to the importance of Na^+^ in Al incorporation, the Na/Al ratio is of particular relevance as it affects the structural integrity of the resulting matrix [16] and the occurrence of efflorescence [17]. Larger Na/Si ratios lead to a higher pH and facilitate Al leaching from the precursor materials [18]. However, they can also result in a surge in sodium ion concentration. According to Degefu et al. [16], ratios above one may result in the continuous diffusion of the alkali ions, and a ratio of 1.2 results in the low durability of the sample. The high sodium concentration can lead to an accumulation of ions on the surface of the samples, which leads to its carbonation due to the exposure of the alkalis to atmospheric conditions [17]. This efflorescence phenomenon reduces the durability and quality of the material [17].

Conversely, lower Na/Al ratios result in a higher amount of bridging Al(OH)^4−^ in the gel and a larger mean chain length [17], integrating more Na^+^ ions in the matrix, which promotes a chemically steadier sodium species [17]. However, an excess of Al_2_O_3_ can also cause a pH decrease, which influences the dissolution of the precursor and the polymerization of the hydration products [16]. Thermodynamic models developed by Yan et al. [19] highlight the importance of the alkalinity of the mixture, as the surge of alkali hydroxide increases the concentration of Al and Si in solution, since, with the pH increase, there is a preference for Al to form Al(OH)^4−^ complexes and Si to form SiO(OH)_3_^−^ and SiO_2_(OH)_2_^2−^ [20].

Other characteristics that influence properties such as the chain length [14] and morphology [21] of the hydration product are the Ca/(Si + Al), Si/Al, and Si/Na ratios [21,22]. Hence, due to the high sensitivity of the system to these ratios, when alkaline activation is required, it is important to consider both the binder and activator’s chemical composition [7,12,14,23,24,25,26,27,28,29,30,31].

Notably, sodium silicate (SS) solution has been shown to be an effective activator, giving rise to fast hardening and high compressive strength results [10,32,33,34]. Furthermore, the use of this activator has been correlated to a higher Al integration in tetrahedra position within the C-A-S-H gel and a higher intertwining between chains [14]. Two variables that strongly influence the performance of this activator are the SiO_2_/Na_2_O MS ratio, which impacts the amount of soluble Si content in the system [32,35], influencing the dissolution and absorption of the species [22], and the Na_2_O molar concentration, which affects the alkalinity of the solution [36] and the erosion of the grains [22]. Hence, a balance between these two parameters allows better strength results.

Previous research focused on the impact of the MS ratio on the properties of the materials [27,37,38,39]. Sun et al. [38] studied the influence of MS ratio ranging from 0.5 to 2.0 in pastes. The results indicated that an increase in MS ratio prolonged the setting time, reduced workability, and refined the pore structure of the activated material. Similarly, Caron et al. [40] characterized slag pastes activated with an MS ratio of 0.5 and 2.2 and reported that higher modulus led to slower dissolution rates and a lower C/S ratio in the final product. Additionally, Cihangir et al. [39] reported that for slag activation, MS values of 1.0–1.25 resulted in higher compressive strength at both short and later ages; moreover, an increase of the ratio promoted the formation of a more condensed and polymerized C-S-H structure. The findings of Aydin et al. [27] indicated that an increase in MS ratio led to microcrack formation in the matrix, while low modulus results in higher compressive strengths at early ages.

Even though the MS ratio has been extensively investigated, there is still a need to understand how the available silicate added by the activator influences the hydration reaction of the mixture. Moreover, the effect of increased alumina content in the precursor activation requires further research. Hence, this phenomenon and the effects of the MS ratio of the activator and the Al_2_O_3_ content of the sample on the hydration reaction and paste performance were investigated. This study allows the optimization of the activator of a specific precursor and, consequently, improves the performance of the material.

In this work, the influence of increasing the Al_2_O_3_ content up to 12%wt on the AWH was tested on alkaline-activated pastes. A sodium silicate solution was used as the activator, and its composition was optimized in terms of MS ratio and Na_2_O molarity. The hydration reaction was followed using as isothermal calorimeter, and the performance of the pastes was tested at 2, 7, and 28 days by applying compressive strength to the sample. Each sample was then analyzed by Fourier-transform infrared spectroscopy (FTIR), X-ray diffraction (XRD), and thermogravimetric analysis (TGA).

## 2. Materials and Methods

### 2.1. Binder Production

The binder production procedure was similar to previous studies [10,41,42,43]. In this work, four AWH binders were produced using common OPC clinker raw materials (limestone, marl, sand, and fuel cracking catalyst waste to adjust Al_2_O_3_ content). The chemical composition was adjusted so that the overall C/S molar ratio was of ~1.0 and the theoretical %wt Al_2_O_3_ content was of 4%, 7%, 10%, and 12%. The chemical composition of the four binders is presented in Table 1.

The clinker of each binder was produced using the same process treatment. First, the raw materials were ground, mixed, and compressed in discs to facilitate their introduction to a silicon carbide crucible. The filled crucible was heated in an electric furnace using the following steps: (1) increase temperature to 900 °C at 25 °C/min rate; (2) the 900 °C temperature was maintained for 1 h to ensure full decarbonation; (3) increase temperature to 1550 °C at 25 °C/min; and (4) the temperature was maintained at 1550 °C for 1 h to allow for complete melting and chemical homogenization. Finally, the resultant material was quenched into a container filled with water.

The AWH clinker produced was dried at 100 °C for 1 h to remove the water from the quenching process and ground for 3 min using a ring mill, obtaining a Blaine of approximately 6000 cm^2^/g and a 45 µm residue below 20%wt.

### 2.2. Production of the Pastes: Activation Conditions

The pastes were hydrated using an Na_2_SiO_3_ + NaOH activator with a water/solid ratio of 0.3. The alkaline solution was prepared using a standard sodium silicate solution, Na_2_O: 7.5–8.5%; SiO_2_: 25.5–28.5% (Chem Lab, Zedelgem, Belgium) equilibrated with NaOH pebbles, 98.2% (Prolabo,, Matsonford, PA, USA). The prepared pastes were introduced into molds with a 20 × 20 × 40 mm^3^ dimension and cured at 20 °C under relative humidity conditions over 90%. After 2 days of curing, the samples were removed from the molds and left to cure under the same conditions.

Three sodium silicate activators with different conditions, MS ratio, and Na_2_O molarity were used to activate each sample. The characteristic of each solution is displayed in Table 2.

### 2.3. Isothermal Calorimetry

The hydration reaction of the pastes was followed using an isothermal calorimeter (TAM Air instrument, Waters Sverige AB, Sollentuna, Sweden). The tests were performed using a constant temperature of 20 °C.

### 2.4. Compressive Strength Tests

To assess the pastes’ performance after 2, 7, and 28 days of curing, compressive strength tests were performed in an Ibertest Autotest 400/10 instrument using a constant force rate of 2.4 kN/s. The resultant paste debris was ground by hand and dried at 100 °C to remove evaporable water and to avoid further hydration. The samples were then characterized by FTIR and XRD.

### 2.5. FTIR Analyses

The FTIR-ATR analysis was performed on a bench-top Bruker, model ALPHA, operating with a Platinum ATR module, with the following conditions: 4 cm^−1^ resolution; 24 scans.

### 2.6. XRD Analyses

The X-ray diffraction analysis was performed in an X’Pert Pro (PANalytical, Tokyo, Japan) diffractometer using monochromatic CuKα1 radiation (λ = 1.54059 Å) and working in reflection geometry (θ/2θ). The X-ray tube worked at 45 kV and 40 mA. The configuration used was the following: optics configuration fixed divergence slit (1/2°), a fixed incident anti-scatter slit (1°), fixed diffracted anti-scatter slit (1/2°), and X’Celerator RTMS (Realtime Multiple Strip) detectors, working in scanning mode with maximum active length. For all samples, the data were collected from 5° to 70° (2θ). To enhance particle statistics, the samples were rotated during data collection at 16 rpm.

### 2.7. Thermogravimetric Analysis

The TGA analysis was performed in an ELTRA equipment. The heating rate of the test was fixed at four temperature intervals, 105 °C, 250 °C, 500 °C, and 950 °C, and maintained until a stable mass was reached. The initial step (room temperature–105 °C) was performed at a 4 °C/min rate in order to remove evaporable water that could remain in the sample, the second step (105–250 °C) was performed at 10 °C/min rate, and the third and fourth steps (250–500 °C and 500–900 °C) were performed at a 15 °C/min a rate. The mass loss from the 105–250 °C and 250–500 °C steps was used to estimate the amount of bound water in each paste.

## 3. Results

### 3.1. Isothermal Calorimetry

**Figure 1:**
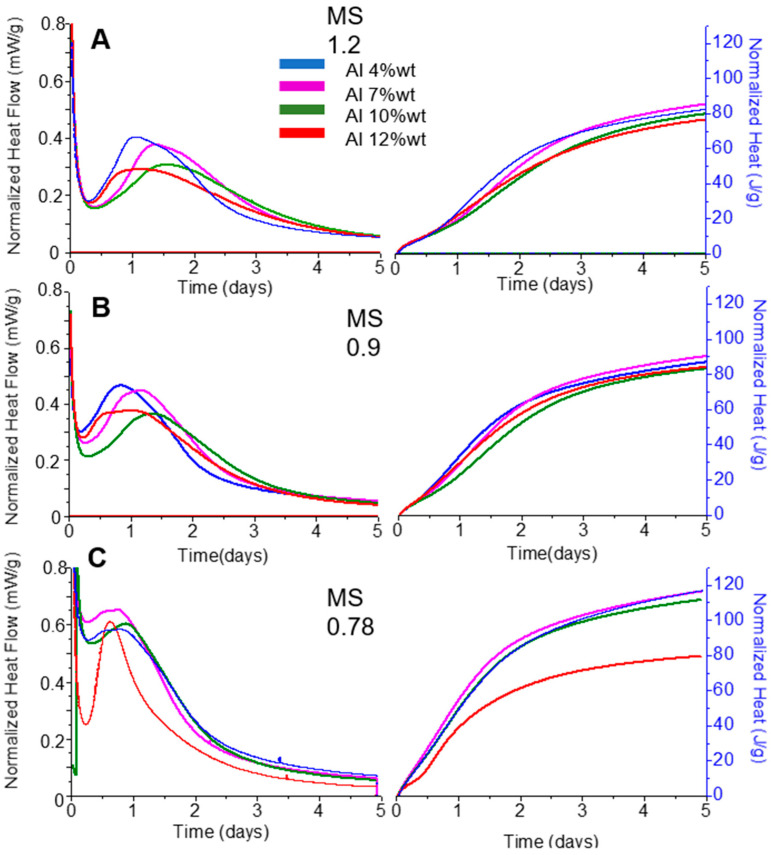
Comparison of isothermal calorimetry results of the AWH pastes, varying the Al content (blue line 4%wt, pink line 7%wt, green line 10%wt, and red line 12%wt) and the activating conditions; (**A**) Si/Na of 1.20 (**B**) Si/Na of 0.90, and (**C**) Si/Na of 0.75.

For each sample, the hydration reaction was followed for five days through isothermal calorimetry. The results are displayed in Figure 1.

After five days of hydration, independently of the MS of the activating solution, all Al12%wt samples displayed an accumulated heat of 80 J/g. However, for the samples with lower Al content, 4%, 7%, and 10%wt, the results were influenced by the MS content of the solution. The highest values were obtained by the pastes hydrated with a MS0.75 activator.

The results of the pastes hydrated with the MS0.9 and MS1.2 solutions (Figure 1A,B) indicate that an increase in Al_2_O_3_ content leads to a broadening of the hydration peak and a decrease in its intensity. Moreover, the MS1.2 solution displayed an accumulated heat that ranged from 76 to 85 J/g, while the samples hydrated with the MS0.9 solution ranged from 83 to 90 J/g. This increase of accumulated heat with the reduction of the MS ratio suggests a higher extent of the hydration reaction. Finally, the calorimetric results in Figure 1C show the influence of further reducing the MS ratio to 0.75. All samples displayed an anticipation of the hydration peak, indicating an increase in the kinetic reaction and an increase of heat released after five days of hydration (~110 J/g).

### 3.2. Compressive Strength Results in Pastes

**Figure 2:**
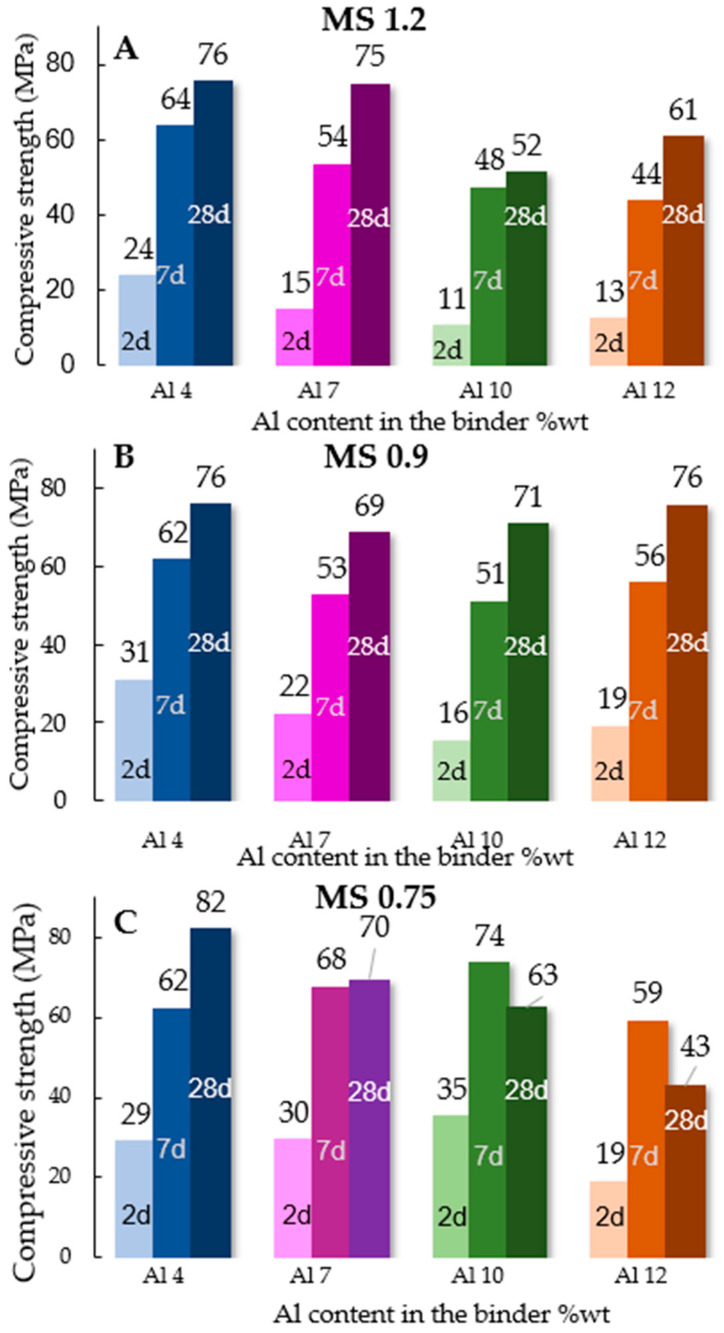
Compressive strength of AWH in relation to the Al content of the binder pastes after 2, 7, and 28 days of hydration. The activating conditions were (**A**) Si/Na of 1.20; (**B**) Si/Na of 0.90, and (**C**) Si/Na of 0.75.

The performance of the produced pastes was tested after 2, 7, and 28 days of hydration through compressive strength tests. The results are presented in Figure 2.

The results indicate that the performance of the pastes is influenced by the binders’ Al_2_O_3_ content, as samples with 4%wt Al_2_O_3_ displayed similar results at all ages regardless of the activator used, while samples with a higher Al_2_O_3_ content exhibited higher susceptibility to the activating solution. Furthermore, when the Na_2_O was increased and the MS ratio was reduced to 0.75, an initial increase in compressive strength at 2 and 7 days of curing was observed, followed by a decrease after 28 days of curing.

### 3.3. FTIR Results

**Figure 3:**
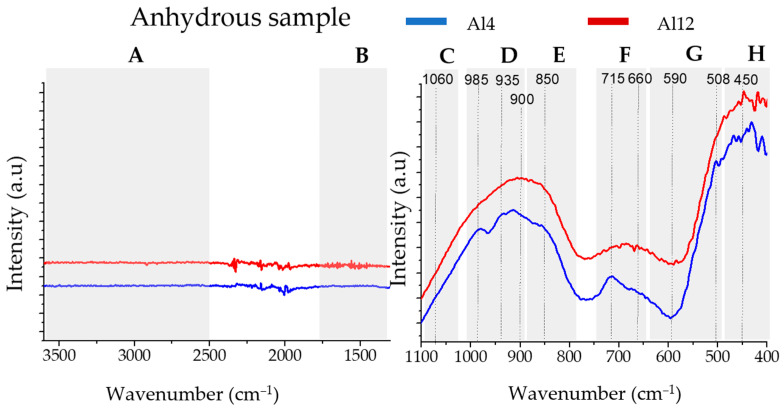
FTIR spectra of the anhydrous Al4% and Al 12% samples (blue and red line, respectively). The spectra were divided into eight main bands, from A to H, for interpretation.

**Figure 4:**
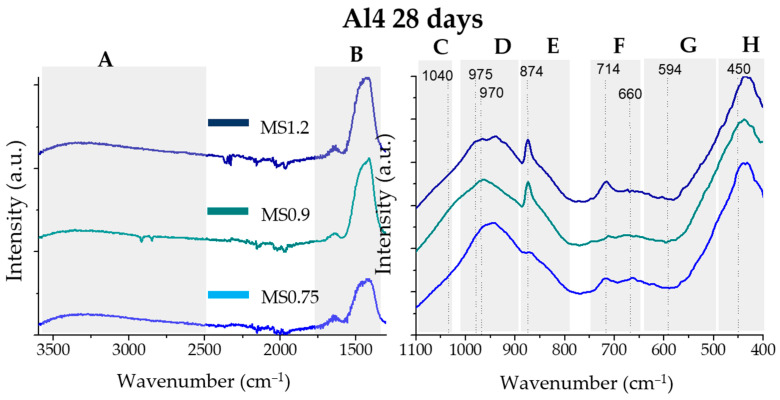
FTIR spectra of the Al4 pastes, activated with the MS075, MS09, and MS1.2 activators (bottom, middle, and top lines, respectively). The spectra were divided into eight main bands, from A to H, for interpretation.

**Figure 5:**
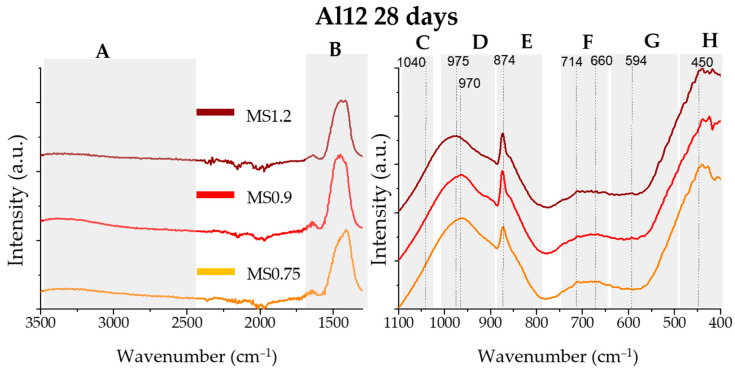
FTIR spectra of the Al12 pastes, activated with the MS075, MS09, and MS1.2 activators (bottom, middle, and top lines, respectively). The spectra were divided into eight main bands, from A to H, for interpretation.

In Figure 3 the FTIR results of the Al4 (blue line) and Al12 (red line) anhydrous samples are displayed. Figure 4 and Figure 5 show the FTIR results for the Al4 and Al12 pastes, respectively, activated with the MS075, MS09, and MS1.2 activators, after 28 days of curing.

The FTIR spectra can be divided into eight main bands, identified as follows:

- 3800–2500 cm^−1^, broad band associated with the O-H stretching [44,45].
- 1700–1300 cm^−1^, CO_3_^2−^ characteristic bands at 1450 cm^−1^ attributed to asymmetric stretching mode [45].
- 1100–1050 cm^−1^, bands characteristic of stretching Si-O-Si bond [44,46,47].
- 1000–900 cm^−1^, the band at ~973 cm^−1^ can be attributed to the Si-O symmetric stretching vibration [45] and the bands at ~980 cm^−1^ [48] and ~900 cm^−1^ [49,50] to the Si-O-Si asymmetric stretching vibration, specifically Q^2^ and Q^1^ structures, respectively.
- 900–800 cm^−1^ the band at 850 cm^−1^ can be attributed to the Si-O-Si asymmetric stretching vibration of Q^0^ units and the 875 cm^−1^ band to the in-plane bending mode of CO_3_^2−^ [10,51,52].
- 750–650 cm^−1^; the band at 680 cm^−1^ can be attributed to the bending motion of oxygen bonds [50], and the band at 712 cm^−1^ is characteristic of the CO_3_^2−^ in-plane bending modes [10,51,52].
- 650–500 cm^−1^, band at 521 cm^−1^ is characteristic of the O-Si-O out-of-plane bending [53] Al-OH bending vibrations at ~590–570 cm^−1^ [54] and bands associated to Si–O–Al–O bonds at 459–572 cm^−1^ [55].
- 500–400 cm^−1^ band associated to silica deformation [48], and band at ~455 cm^−1^ can be associated to the to the Si-O in-plane bending [47].

In both anhydrous samples, there is an absence of bands characteristic of hydration and carbonation. The main difference between these samples can be observed in the D and G bands. In the D band, the Al4 sample displays a sharp band at 985 cm^−1^ and ~900 cm^−1^, characteristic of Si-O stretching vibration at Q^2^ that can be attributed to the pseudowollastonite phase present in this sample [48].

Figure 4 and Figure 5 display the FTIR spectra of the 28-day hydrated pastes for the Al4 and Al12 samples, respectively. All samples exhibit characteristic OH and CO_3_^2−^ bands (A and B areas), indicating the hydration and carbonation of the binder. In all samples, the hydration reaction caused a narrowing and a shift of the C, D, and E bands towards higher wavenumbers, suggesting a higher degree of polymerization. Furthermore, all samples displayed a band at ~970 cm^−1^, typical of the Si–O stretching vibrations in C-S-H gel [48,56]. In the H region, a narrowing of the bands is observed indicating a more organized final structure.

In both hydrated Al4 and Al12 samples’ FTIR spectra, the MS1.2 and MS0.9 samples exhibit the main Si-O symmetric vibrations (area D) at higher wavenumber than the MS075 sample, suggesting an increased degree of polymerization in the hydrated paste.

### 3.4. XRD Results

**Figure 6:**
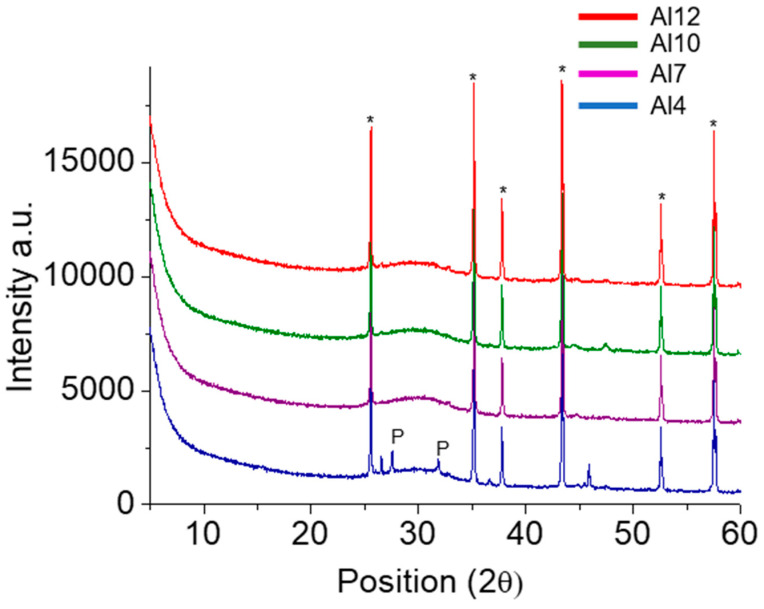
XRD scans of the AWH binders produced with an Al_2_O_3_ wt% content from 4% to 12%. P—pseudowollastonite; *—Corundum, introduced as an internal standard to allow for the calculation of the amorphous wt% content.

The XRD diffractogram and Rietveld analysis of the anhydrous sample produced are displayed in Figure 6 and Table 3, respectively. The results indicate that the Al4 sample displayed the lowest amount of amorphous material (96%) and the presence of pseudowollastonite crystals.

**Figure 7:**
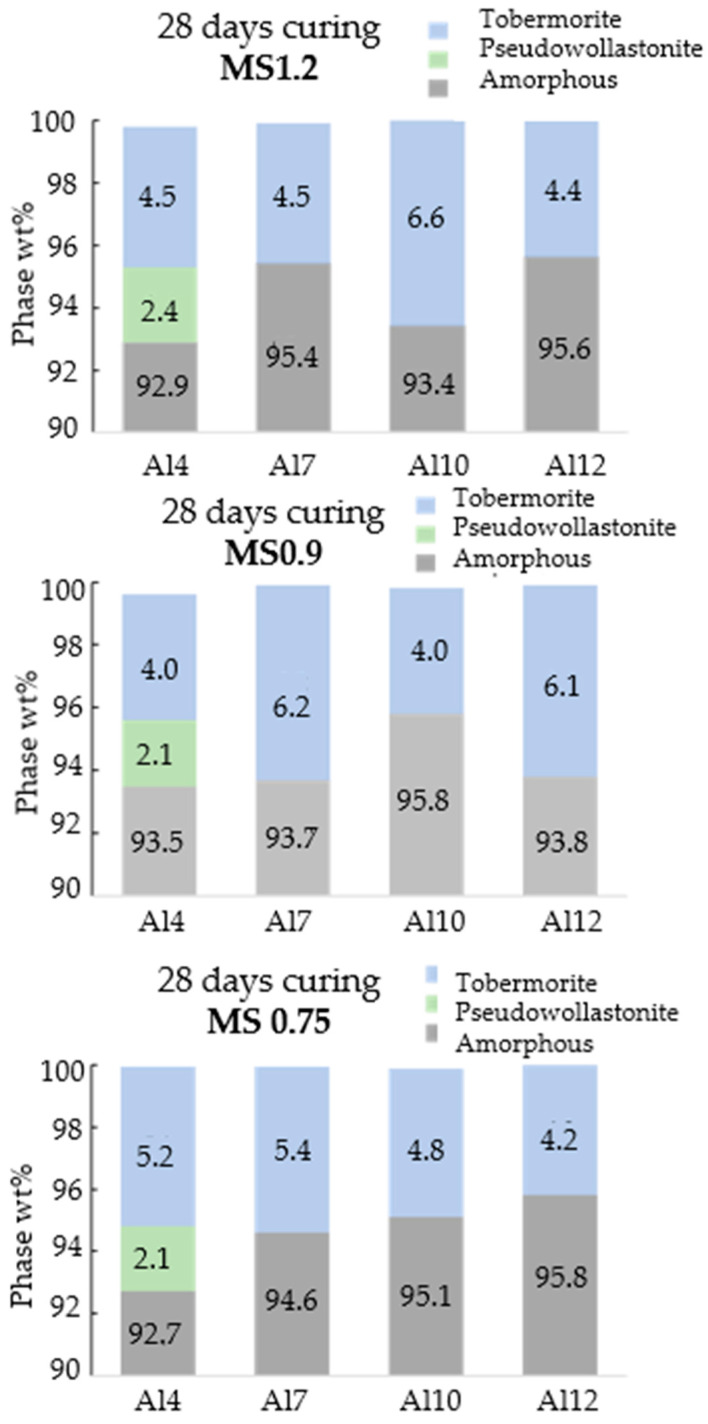
Quantitative results obtained by XRD–Rietveld analysis, in weight percentage, of the phases present in each of the samples studied.

To investigate the phase development after 28-day hydration with different activating solutions, the results obtained through quantitative XRD–Rietveld analysis, in weight percentage, are displayed in Figure 7. The results indicate that all samples, regardless of activation method and Al_2_O_3_ content, exhibited a tobermorite content above 4%wt. Furthermore, the Al4 sample continued displaying a pseudowollastonite content of ~2%wt.

### 3.5. TGA Results

Thermogravimetric analysis of the pastes cured for 2.7 and 28 days was performed in order to calculate the amount of bound water (BW) in the hydrated sample by analyzing the weight loss in the temperature ranges of 105–250 °C and 250–500 °C. In Table 4, the main experimental TGA data obtained is displayed.

**Figure 8:**
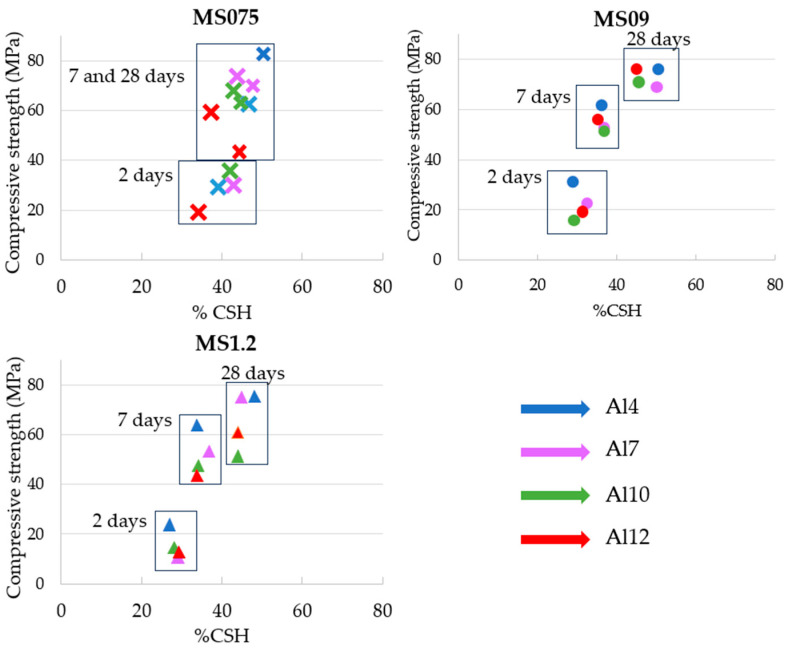
Correlation of compressive strength with the amount of C-S-H formed for the pastes hydrated with an MS075 activator (crosses), an MS09 activator (circles), and an MS1.2 activator (triangles). The binders with an Al_2_O_3_ content (%wt) of 4, 7, 10, and 12 are displayed in blue, purple, green, and red, respectively.

Similarly to previous works [10,41,42,43], the model of Richardson and Qomi [57] and a C/S ratio of 1.1 was assumed, allowing the establishment of a relation between the BW and the amount of C-S-H formed. In Figure 8, a correlation between the %C-S-H calculated and the compressive strength obtained for each sample is displayed.

The obtained results suggest activating the binder with an MS075 solution leads to the rapid formation of C-S-H during the early stages of hydration. However, the formation of hydration products appears to stagnate at later ages. In contrast, the samples hydrated with the MS09 and MS1.2 solutions exhibited lower C-S-H formation at early ages but continuous growth in C-S-H content with curing time.

Moreover, when comparing the amount of C-S-H content with the compressive strength obtained, the results indicate that the pastes activated with the MS075 solution presented a higher content of bound water at the early ages, which correlated well with the higher compressive strength obtained, however, for later ages, the relationship between C-S-H formed and compressive strength seems to differ from the other two conditions tested in this study (MS09 and MS1.2), especially due to the decrease in compressive strength at 28 days for the samples Al10 and Al12 under MS075 activation.

## 4. Discussion

In this work, four AWH clinkers were produced with different Al_2_O_3_ wt% contents: 4, 7, 10, and 12%wt. Each anhydrous clinker was analyzed by XRD–Rietveld phase quantification, revealing a high amorphous content (from 96 to 99%). The clinker with the lowest Al_2_O_3_ content exhibited the lowest amount of amorphous material (96%) and evidenced the presence of pseudowollastonite crystals. This phenomenon may be attributed to the fact that, for this system, alumina acts as a melting agent; hence, the clinkers with lower alumina content present higher melting temperatures. Since all samples were melted at the same temperature (1550 °C), the melting of the lowest alumina content sample shows a higher viscosity and density, which may influence the quenching process and promote the formation of pseudowollastonite crystals.

Each binder was activated with three different sodium silicate solutions with MS ratios of 1.2, 0.9, and 0.75, and the hydration reaction of each sample was followed by calorimetry. Comparing the results of the Al4, Al7, and Al10 samples, an increase in the induction period for higher Al_2_O_3_ content is observed. This result may be attributed to the reduced dissolution of [SiO_4_]^4−^ in the presence of Al_2_O_3_ in the solution [58].

Moreover, the results indicate that the MS ratio and Na_2_O molarity of the activator influence the heat release profiles of the hydrated sample. With the decrease in MS ratio, a more extensive hydration reaction and an increase in kinetics are promoted.

The Al12 paste activated with an MS075 solution displayed a significantly longer induction period compared to the other samples hydrated with the same activator. This extended induction period may be a result of the high Al(OH)_4_^−^content in the solution, which delays Si dissolution [58] and, consequently, the reaction. This result suggest that, conversely to the other samples, the increase in Si undersaturation promoted by the high pH and low MS ratio was insufficient to compensate for the increased Al(OH)_4_^−^ content in the solution.

Analyzing the 28-day pastes’ compressive strength results in Figure 2, it is possible to see that the pastes activated with the MS1.2 solution present a decrease in strength with the increase in Al_2_O_3_ content, with a minimum point on the Al10 sample. The compressive strength of the pastes activated with this solution ranged from 52 to 76 MPa. Conversely, samples hydrated with an MS0.9 activator showed a narrower range of results, from 69 to 76 MPa. Hence, when the binder is activated with the MS0.9 solution, the influence of the Al_2_O_3_ content on the sample’s performance seems to be mitigated.

The decrease of the MS ratio to 0.75 led to higher hydration kinetics and high compressive strength results at early ages in the samples with an Al_2_O_3_ content higher than 7%wt. However, after 28 days of curing, a decrease in compressive strength was observed. These results may be a consequence of the high NaOH molarity in solution. At early ages, the increase of pH facilitates the solubility of [SiO_4_]^4−^ ions from the binder [59], promoting the hydration reaction. Moreover, the increase of Na_2_O also allows a higher aluminum incorporation into the C-S-H gel, since the sodium cations are able to stabilize the silicon–aluminum exchange. However, the rapid reaction of the system can lead to a localized increase in the matrix density, resulting in an overall more porous microstructure [11]. Moreover, the high aluminum incorporation leads to a higher extent of formation of C-A-S-H structures to the detriment of C-S-H. Since the compressive strength of C-A-S-H is lower than that of C-S-H, this leads to a reduction of compressive strength [60]

**Figure 9:**
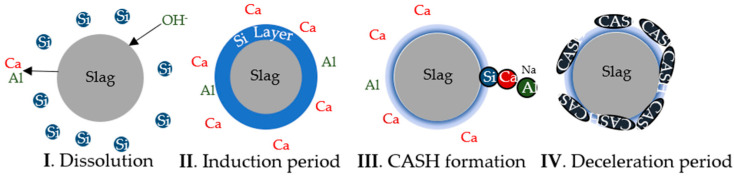
Hydration reaction process of a slag activated with an SS solution with a high MS ratio.

Similar to the activation of blast furnace slag (BFS) [22,61], the results of this study indicate that the SS activation of the AWH binder can be categorized into four main stages, as displayed in Figure 9.

In stage (I), the introduction of free Si species from the activator promotes a very low undersaturation of this species, causing a low Si dissolution from the binder [61]. Conversely, while Si ions remain at the grain surface, the undersaturation of Ca and Al is high, resulting in a release of these elements into the solution [61,62].

In stage (II), the surge of Si in the grains’ surface gradually increases the thickness of the Si layer [22,61]. Hence, while the dissolution of Ca and Al is fast, the Si dissolution is very slow. This stage is associated with the formation of an induction period [22,61,62].

In stage (III), the Ca and Al in solution react with Si, forming a C-A-S-H gel [22]. With the consumption of these ions, the Si undersaturation increases, promoting the dissolution of the Si layer [22,61].

Finally, in stage (IV), the accumulated reaction products inhibit the further dissolution of unreacted particles, leading to a deceleration period. [22,61,62].

In systems with a high MS ratio, the high [SiO_4_]^4+^ concentration can result in the formation of H_2_SiO_3_ structures [22], which, when oversaturated, crystallize as a hydrated sodium metasilicate [22] and result in the precipitation of a gel [63]. Moreover, high MS ratios promote the absorption of Ca^+^ and Al^3+^ by the soluble [SiO_4_]^4+^ [22], leading to a higher degree of polymerization, a higher extent of reaction products [22,37], and a denser microstructure [22,37]. However, this denser structure around the grains can also prevent further dissolution [22], inhibiting the hydration reaction [22] and consequently decreasing the performance of the hydrated material at later ages [64]. Moreover, the excess of [SiO_4_]^4−^ can also promote SiO_2_ precipitation [22].

**Figure 10:**
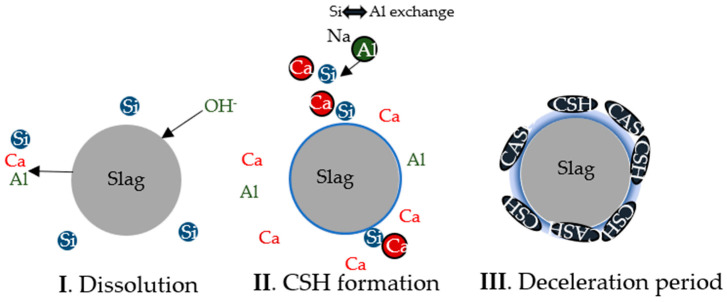
Hydration reaction process of a slag activated with an SS solution with a low MS ratio.

The decrease of the MS ratio is associated to a higher pH, a faster dissolution, a shorter setting time, and a higher flowability [35,64]. As the MS ratio decreases, the [SiO_4_]^4+^ undersaturation increases, promoting the release of Ca^+^, Al^3+^, and [SiO_4_]^4−^ ions into the solution during the dissolution step and the production of a C-S-H gel on the particles’ surface [22]. As the reaction continues, the Al^3+^ replaces the Si^4+^ in the structure [14], and the Na^+^ is incorporated to balance the C-A-S-H gel formed [14]. The increase of the C-S-H/C-A-S-H layer decreases the OH^−^ diffusion into the particle and the Ca^+^ and [SiO_4_]^4−^ diffusion into the solution [22], leading to a deceleration period. In Figure 10, a schematic representation of this mechanism is displayed.

Therefore, the results of this study indicate that to allow the formation of an organized C(A)SH structure, the presence of a Si layer is required. A very thin layer, promoted by a high Si undersaturation (e.g., activation with a MS075 solution), leads to an increase in kinetics and the rapid formation of hydration products. However, this rapid growth inhibits dissolution at later ages and increases the porosity of the hydrated product. Both phenomena result in a decrease in compressive strength at 28 days of hydration.

Conversely, if the layer is too thick, the induction period increases due to the low silica solubility. The low Si undersaturation may compromise the formation of C-S-H products, decreasing the performance of the binder. This is the case for systems with a high Al_2_O_3_ content and activated with a high MS ratio sodium silicate solution.

TGA results confirm this trend; compared to the other solutions, the samples hydrated with the MS075 solution resulted in a higher number of C-S-H structures at early ages. However, the formation of hydration products appears to stagnate at later ages. In contrast, the samples hydrated with the MS09 and MS1.2 solutions lead to a steady increase in hydration products with curing time, particularly in the low-Al_2_O_3_-content samples.

In conclusion, obtaining an equilibrium in Si saturation–undersaturation is fundamental in this system. The Si solubility must be low enough to promote the formation of a Si layer and allow a controlled kinetic and the formation of an organized CS(A)H structure and must be high enough to promote the dissolution of the Si layer and allow the hydration reaction to continue. An increase of Al(OH)_4_^−^ content in solution and an increase of the MS ratio increase the Si saturation, decreasing its solubility, delaying the reaction, and inhibiting the formation of hydration product. Nevertheless, by reducing the MS ratio to 0.9, the Si solubility increases and compensates for the negative effect of Al(OH)_4_^−^, while still maintaining a controlled reaction kinetic.

Therefore, a decrease of the MS ratio from 1.2 to 0.9 led to a more extensive hydration reaction, which translated into higher compressive strength results at later ages. Additionally, in all cases, the presence of sodium is fundamental for Al incorporation in the structure.

## 5. Conclusions

In this work, the influence of the activator on the hydration reaction of AWH samples with different Al_2_O_3_ contents was studied by decreasing the MS modulus from 1.2 to 0.75 and increasing the Na_2_O molarity. Moreover, the performance in pastes was investigated.

The main results indicate that the kinetic of the system is influenced by silica undersaturation in solution, which decreases for higher Al_2_O_3_ content and MS ratio in solution. In systems where silica undersaturation is too low, the kinetic of the mixture is delayed, and the formation of more hydration products at later ages is inhibited due to the low Si solubility. Conversely, if the silica undersaturation is excessively high, it promotes the uncontrolled rapid growth of calcium silicate hydrate structures (C-S-H), which can compromise the increase of compressive strength at later ages.

For this specific binder composition, the experimental results suggest that an activator with an MS ratio of 0.9 and a Na_2_O molarity of 3.516M leads to a more controlled kinetic mixture and overall higher performance, even on binders with a higher Al_2_O_3_ content. Since a further reduction of the MS ratio had a negative impact on the long-term performance of paste, we concluded that a MS0.9 may represent the optimized composition of the activator.

Hence, in this study, the activator was optimized to the precursor, allowing an increase in the range of Al_2_O_3_ content that can be incorporated into the clinker, which could potentially allow a reduction in the melting temperature of the material.

## Figures and Tables

**Table 1:** Chemical composition of the produced binders.

%wt	Al_4%	Al_7%	Al_10%	Al_12%
SiO_2_	47.51	45.74	44.58	43.09
Al_2_O_3_	3.74	6.52	9.67	12.49
Fe_2_O_3_	0.58	1.14	1.02	1.07
CaO	46.04	44.47	42.73	40.3
MgO	0.67	0.62	0.57	0.52
Other elements	<1.50	<1.50	<1.50	<1.50
C/S (molar)	1.04	1.04	1.03	1.00

**Table 2:** Nomenclature of the sodium silicate solutions used as activators.

Nomenclature	Si/Na	Na_2_O (M)	pH
MS1.2	1.20	3.52	13.3
MS0.9	0.90	3.52	13.3
MS0.75	0.75	4.92	13.8

**Table 3:** Rietveld analysis of the AWH binders produced with variable Al_2_O_3_ wt% content from 4% to 12%.

AWH Binder Al_2_O_3_%	Pseudowollastonite	Wollastonite	Amorphous
Al4	3.2	0.15	96.65
Al7	0	0.2	99.8
Al10	0	0.3	99.7
Al12	0	0.2	99.8

**Table 4:** TGA experimental results obtained for the studied specimens. LOI indicates the loss in ignition at specific temperature steps. Values are shown in weight percentages.

		2 Days			7 Days			28 Days		
Samples		FL 105–250 °C	FL 250–500 °C	FL 500–950 °C	FL 105–250 °C	FL 250–500 °C	FL 500–950 °C	FL 105–250 °C	FL 250–500 °C	FL 500–950 °C
MS 0.75	Al4	2.67	1.56	0.08	2.46	2.60	0.46	2.33	3.05	0.57
	Al7	2.80	1.84	0.08	2.42	2.21	0.71	2.98	2.16	0.88
	Al9	2.51	2.03	0.11	2.08	2.66	0.56	2.47	2.35	0.68
	Al12	2.32	1.40	0.09	2.29	1.75	0.44	2.56	2.20	0.88
MS 0.9	Al4	1.90	1.23	0.20	2.67	1.24	0.19	2.47	2.94	0.38
	Al7	2.19	1.33	0.27	2.65	1.34	0.40	2.54	2.61	1.95
	Al9	2.01	1.17	0.25	2.15	1.83	0.54	2.47	2.32	1.13
	Al12	2.14	1.26	0.39	2.18	1.63	0.40	2.38	2.31	0.74
MS 1.2	Al4	1.88	1.02	0.22	2.45	1.18	0.24	2.32	4.09	0.49
	Al7	1.97	1.07	0.31	2.38	1.57	0.88	3.06	2.33	1.40
	Al9	2.02	1.12	0.21	2.15	1.54	0.89	2.25	2.64	2.80
	Al12	1.85	1.31	0.36	2.24	1.40	0.68	2.45	2.39	2.16

## Data Availability

Data are contained within the article.

## References

[1] European Commission, Directorate-General for Internal Market, Industry, Entrepreneurship, SMEs (2017). Competitiveness of the European Cement and Lime—Final Report.

[2] Supino S., Malandrino O., Testa M., Sica D. (2016). Sustainability in the EU Cement Industry: The Italian and German Experiences. J. Clean. Prod..

[3] Scrivener K.L., John V.M., Gartner E.M. (2018). Eco-Efficient Cements: Potential Economically Viable Solutions for a Low-CO_2_ Cement-Based Materials Industry. Cem. Concr. Res..

[4] Freitas A.A., Santos R.L., Colaço R., Bayão Horta R., Canongia Lopes J.N. (2015). From Lime to Silica and Alumina: Systematic Modeling of Cement Clinkers Using a General Force-Field. Phys. Chem. Chem. Phys..

[5] Chappex T., Scrivener K.L. (2012). The Influence of Aluminium on the Dissolution of Amorphous Silica and Its Relation to Alkali Silica Reaction. Cem. Concr. Res..

[6] Rahman A., Ekaputri J.J. The Effect of Additional Aluminium to the Strength of Geopolymer Paste. Proceedings of the 4th International Conference on Rehabilitation and Maintenance in Civil Engineering (ICRMCE 2018).

[7] Jan A., Pu Z., Khan K.A., Ahmad I., Shaukat A.J., Hao Z., Khan I. (2022). A Review on the Effect of Silica to Alumina Ratio, Alkaline Solution to Binder Ratio, Calcium Oxide + Ferric Oxide, Molar Concentration of Sodium Hydroxide and Sodium Silicate to Sodium Hydroxide Ratio on the Compressive Strength of Geopolymer Concrete. Silicon.

[8] Horta R.S.B., Colaço R.A.C., Lopes J.N.A., Santos R.L., Pereira J.C., Rocha P.J.P., Lebreiro S.M.M. (2015). Amorphous Low-Calcium Content Silicate Hydraulic Binders and Methods for Their Manufacturing. U.S. Patent.

[9] Santos R.L., Horta R.B., Pereira J., Nunes T.G., Rocha P., Lopes J.N.C., Colaço R. (2016). Novel High-Resistance Clinkers with 1.10<CaO/SiO_2_<1.25 : Production Route and Preliminary Hydration Characterization. Cem. Concr. Res..

[10] Santos R.L., Horta R.B., Al E. (2018). Alkali Activation of a Novel Calcium-Silicate Hydraulic Binder. Am. Ceram. Soc..

[11] Winnefeld F., Haha B., Le Saout G., Costoya M., Ko S.-C., Lothenbach B. (2015). Influence of Slag Composition on the Hydration of Alkali-Activated Slags. J. Sustain. Cem. Mater..

[12] Haha M.B., Lothenbach B., Le Saout G., Winnefeld F. (2012). Influence of Slag Chemistry on the Hydration of Alkali-Activated Blast-Furnace Slag—Part II: Effect of Al_2_O_3_. Cem. Concr. Res..

[13] Renaudin G., Russias J., Leroux F., Frizon F., Cau-dit-Coumes C. (2009). Structural Characterization of C-S-H and C-A-S-H Samples-Part I: Long-Range Order Investigated by Rietveld Analyses. J. Solid State Chem..

[14] Puertas F., Palacios M., Manzano H., Dolado J.S., Rico A., Rodríguez J. (2011). A Model for the C-A-S-H Gel Formed in Alkali-Activated Slag Cements. J. Eur. Ceram. Soc..

[15] Pardal X., Brunet F., Charpentier T., Pochard I., Nonat A. (2012). ^27^Al and ^29^Si Solid-State NMR Characterization of Calcium-Aluminosilicate-Hydrate. Inorg. Chem..

[16] Degefu D.M., Berardi U. (2024). Effect of Na/Al and Curing Moisture Conditions on Sodium Aluminosilicate Hydrate (N–A–S–H) Geopolymers’ Hydric Properties. Constr. Build. Mater..

[17] Liu T., Chen Y., Yuan B., Zhuang W., Brouwers H.J.H., Yu Q. (2024). Sodium Aluminate Activated Waste Glass: Reduced Efflorescence Behavior by C(N)–A–S–H Transformation. Cem. Concr. Res..

[18] Yaakob S.M., Rabat N.E., Sufian S. (2018). Effects of Na: Al and Water: Solid Ratios on the Mechanical Properties of Fly Ash Based Geopolymer. IOP Conf. Ser. Mater. Sci. Eng..

[19] Yan Y., Ma B., Miron G.D., Kulik D.A., Scrivener K., Lothenbach B. (2022). Al Uptake in Calcium Silicate Hydrate and the Effect of Alkali Hydroxide. Cem. Concr. Res..

[20] Leonelli C., Palomo Á., Luukkonen T., Luukkonen T. (2022). Alkali-Activated Materials in Environmental Technology.

[21] Zhu X., Richardson I.G. (2023). Morphology-Structural Change of C-A-S-H Gel in Blended Cements. Cem. Concr. Res..

[22] Sun B., Ye G., de Schutter G. (2022). A Review: Reaction Mechanism and Strength of Slag and Fly Ash-Based Alkali-Activated Materials. Constr. Build. Mater..

[23] Ashraf W. (2018). Microstructure of Chemically Activated of Gamma-Dicalcium Silicate Paste. Constr. Build. Mater..

[24] Yan Y., Yang S.Y., Miron G.D., Collings I.E., L’Hôpital E., Skibsted J., Winnefeld F., Scrivener K., Lothenbach B. (2022). Effect of Alkali Hydroxide on Calcium Silicate Hydrate (C-S-H). Cem. Concr. Res..

[25] Mierzwiński D., Walter J., Olkiewicz P. The Influence of Alkaline Activator Concentration on the Apparent Activation Energy of Alkali-Activated Materials. Proceedings of the MATBUD’2020—Scientific-Technical Conference: E-Mobility, Sustainable Materials and Technologies.

[26] Bondar D., Lynsdale C.J., Milestone N.B., Hassani N., Ramezanianpour A.A. (2011). Effect of Type, Form, and Dosage of Activators on Strength of Alkali-Activated Natural Pozzolans. Cem. Concr. Compos..

[27] Aydın S., Baradan B. (2014). Composites: Part B Effect of Activator Type and Content on Properties of Alkali-Activated Slag Mortars. Compos. Part B Eng..

[28] Ben Haha M., Le Saout G., Winnefeld F., Lothenbach B. (2011). Influence of Activator Type on Hydration Kinetics, Hydrate Assemblage and Microstructural Development of Alkali Activated Blast-Furnace Slags. Cem. Concr. Res..

[29] Puertas F. (2017). Alkaline Activation of Different Aluminosilicates as an Alternative to Portland Cement: Alkali Activated Cements or Geopolymers. Rev. Ing. Constr..

[30] Abdullah M.M.A., Kamarudin H., Mohammed H., Khairul Nizar I., Rafiza A.R., Zarina Y. (2011). The Relationship of NaOH Molarity, Na_2_SiO_3_/NaOH Ratio, Fly Ash/Alkaline Activator Ratio, and Curing Temperature to the Strength of Fly Ash-Based Geopolymer. Adv. Mat. Res..

[31] França S., de Moura Solar Silva M.V., Ribeiro Borges P.H., da Silva Bezerra A.C. (2022). A Review on Some Properties of Alkali-Activated Materials. Innov. Infrastruct. Solut..

[32] Wang S.-D., Scrivener K.L., Pratt P.L. (1994). Factors Affecting the Strength of Alklai-Activated Slag. Cem. Concr. Res..

[33] Nodehi M., Taghvaee V.M. (2022). Alkali-Activated Materials and Geopolymer: A Review of Common Precursors and Activators Addressing Circular Economy. Circ. Econ. Sustain..

[34] Brough A.R., Atkinson A. (2002). Sodium Silicate-Based, Alkali-Activated Slag Mortars—Part I. Strength, Hydration and Microstructure. Cem. Concr. Res..

[35] Luukkonen T., Sreenivasan H., Abdollahnejad Z., Yliniemi J., Kantola A., Telkki V.V., Kinnunen P., Illikainen M. (2020). Influence of Sodium Silicate Powder Silica Modulus for Mechanical and Chemical Properties of Dry-Mix Alkali-Activated Slag Mortar. Constr. Build. Mater..

[36] Luukkonen T., Abdollahnejad Z., Yliniemi J., Kinnunen P., Illikainen M. (2018). Cement and Concrete Research One-Part Alkali-Activated Materials: A Review. Cem. Concr. Res..

[37] Cho Y.K., Yoo S.W., Jung S.H., Lee K.M., Kwon S.J. (2017). Effect of Na_2_O Content, SiO_2_/Na_2_O Molar Ratio, and Curing Conditions on the Compressive Strength of FA-Based Geopolymer. Constr. Build. Mater..

[38] Sun J., Hou S., Guo Y., Wei H., Jiuwen B., Yifei C., Peng Z. (2024). Sustainable Utilization of Alkali-Activated Steel Slag Material: Effects of Silicate Modulus and GBFS on Fresh, Mechanical and Pore Structure Properties. Dev. Built Environ..

[39] Cihangir F., Ercikdi B., Kesimal A., Ocak S., Akyol Y. (2018). Effect of Sodium-Silicate Activated Slag at Different Silicate Modulus on the Strength and Microstructural Properties of Full and Coarse Sulphidic Tailings Paste Backfill. Constr. Build. Mater..

[40] Caron R., Patel R.A., Miron G.D., Le Galliard C., Lothenbach B., Dehn F. (2023). Microstructure Development of Slag Activated with Sodium Silicate Solution: Experimental Characterization and Thermodynamic Modeling. J. Build. Eng..

[41] Antunes M., Santos R.L., Pereira J., Horta R.B., Colaço R. (2024). The Use of Solid Sodium Silicate as Activator for an Amorphous Wollastonitic Hydraulic Binder. Materials.

[42] Antunes M., Santos R.L., Horta R.B., Colaço R. (2023). Novel Amorphous-Wollastonitic Low-Calcium Hydraulic Binders: A State-of-the-Art Review. Materials.

[43] Santos R.L. (2016). New Hydraulic Binders with Low Calcium Content. Ph.D. Thesis.

[44] Ellerbrock R., Stein M., Schaller J. (2022). Comparing Amorphous Silica, Short-Range-Ordered Silicates and Silicic Acid Species by FTIR. Sci. Rep..

[45] Husung R.D., Doremus R.H. (1990). The Infrared Transmission Spectra of Four Silicate Glasses before and after Exposure to Water. J. Mater. Res..

[46] Pérez-Casas J.A., Zaldívar-Cadena A.A., Álvarez-Mendez A., Ruiz-Valdés J.J., Parra-Arciniega S.M.D.L., López-Pérez D.C., Sánchez-Vázquez A.I. (2023). Sugarcane Bagasse Ash as an Alternative Source of Silicon Dioxide in Sodium Silicate Synthesis. Materials.

[47] Garcia M.D. (2018). Synthesis by Supercritical Fluids Methods of Advanced Additions for Cementitious Materials. Ph.D. Thesis.

[48] Ping Y., Kirkpatrick R.J., Brent P., McMillan P.F., Cong X. (1999). Structure of Calcium Silicate Hydrate (C-S-H): Near-, Mid-, and Far-Infrared Spectroscopy. J. Am. Ceram. Soc..

[49] Lin J., Zhang Y., Yang Z. (2023). A Review of Recent Advances in Alkali-Activated Materials from Silica-Rich Wastes Derived Sodium Silicate Activators. J. Adv. Concr. Technol..

[50] Kalampounias A.G. (2011). IR and Raman Spectroscopic Studies of Sol-Gel Derived Alkaline-Earth Silicate Glasses. Bull. Mater. Sci..

[51] Hughes T.L., Methven C.M., Jones T.G.J., Pelham S.E., Fletcher P., Hall C. (1995). Determining Cement Composition by Fourier Transform Infrared Spectroscopy. Adv. Cem. Based Mater..

[52] Bosch Reig F., Adelantado J.V.G., Moreno M.C.M.M. (2002). FTIR Quantitative Analysis of Calcium Carbonate (Calcite) and Silica (Quartz) Mixtures Using the Constant Ratio Method. Application to Geological Samples. Talanta.

[53] Singh N.B., Das S.S., Singh P., Dwivedi N. (2009). Studies on SCLA Composite Portland Cement. Indian J. Eng. Mater. Sci..

[54] Arioz E., Arioz O., Kockar O.M. (2020). Geopolymer Synthesis with Low Sodium Hydroxide Concentration. Iran. J. Sci. Technol. Trans. Civ. Eng..

[55] Handayani L., Aprilia S., Abdullah, Rahmawati C., Aulia T.B., Ludvig P., Ahmad J. (2022). Sodium Silicate from Rice Husk Ash and Their Effects as Geopolymer Cement. Polymers.

[56] García Lodeiro I., Macphee D.E., Palomo A., Fernández-Jiménez A. (2009). Effect of Alkalis on Fresh C-S-H Gels. FTIR Analysis. Cem. Concr. Res..

[57] Richardson I.G. (2014). Model Structures for C-(A)-S-H(I). Acta Crystallogr. B Struct. Sci. Cryst. Eng. Mater..

[58] Bagheri M., Lothenbach B., Shakoorioskooie M., Scrivener K. (2022). Effect of Different Ions on Dissolution Rates of Silica and Feldspars at High PH. Cem. Concr. Res..

[59] Dupuis R., Rodrigues D.G., Champenois J.B., Pellenq R.J.M., Poulesquen A. (2020). Time Resolved Alkali Silicate Decondensation by Sodium Hydroxide Solution. J. Phys. Mater..

[60] Visser J., Garzon-Amortegui J., Nijland T., Hermanns S. (2023). Microstructure and Performance of Three Silicate Binders in the Range CSH-CASH-NAS. International RILEM Conference on Synergising Expertise towards Sustainability and Robustness of Cement-Based Materials and Concrete Structures.

[61] Zuo Y., Ye G. (2020). Preliminary Interpretation of the Induction Period in Hydration of Sodium Hydroxide/Silicate Activated Slag. Materials.

[62] Sun X., Zhao Y., Qiu J., Xing J. (2022). Review: Alkali-Activated Blast Furnace Slag for Eco-Friendly Binders. J. Mater. Sci..

[63] Provis J.L., Duxson P., Lukey G.C., Separovic F., Kriven W.M., Van Deventer J.S.J. (2005). Modeling Speciation in Highly Concentrated Alkaline Silicate Solutions. Ind. Eng. Chem. Res..

[64] Ouyang X., Ma Y., Liu Z., Liang J., Ye G. (2019). Effect of the Sodium Silicate Modulus and Slag Content on Fresh and Hardened Properties. Minerals.

